# Up-to-date earthquake and focal mechanism solutions datasets for the assessment of seismic hazard in the vicinity of the United Arab Emirates

**DOI:** 10.1016/j.dib.2019.104844

**Published:** 2019-11-20

**Authors:** Rashad Sawires, José A. Peláez, Mohammad AlHamaydeh, Jesús Henares

**Affiliations:** a Department of Geology, Faculty of Science, Assiut University, PO Box 71516, Assiut, Egypt; b Institute of Geophysics - Morelia Campus, National Autonomous University of Mexico (UNAM), PO Box 58190, Morelia, Michoacán, Mexico; c Department of Physics, University of Jaén, PO Box 23071, Jaén, Spain; d Civil Engineering Department, American University of Sharjah, PO Box 26666, Sharjah, United Arab Emirates; e International University of La Rioja, PO Box 26006, Logroño, Spain

**Keywords:** Earthquake catalog, Focal mechanism solutions, Moment magnitude, Seismic hazard, United Arab Emirates

## Abstract

This article presents updated and unified Poissonian earthquake and focal mechanism catalogs for the United Arab Emirates and its surroundings. Data were compiled from different local, regional, and international sources. Several magnitude-conversion relationships have been applied to obtain the unified moment magnitude estimates for the final datasets. Dependent earthquakes were identified and removed from the compiled data via a declustering process to ensure a time independent Poissonian distribution of seismicity. The final compiled earthquake catalog is comprised of 1464 mainshocks, which span the time period of 658–2019 [1]. The range of their spatial region is 21°–31°N on the latitude and 47° to 66°E on the longitude. Additionally, an overall number of 583 focal mechanism solutions spanning the time period of 1923–2015 were acquired [1]. Such datasets are compatible with other published catalogs from across the globe which provide a basis for the estimation of seismic hazard and risk, as well as, the establishment of a unified seismic action representation in the building codes for the United Arab Emirates. This paper and its dataset are a companion for a published article in the Journal of Asian Earth Sciences under the title “A State-Of-The-Art Seismic Source Model for the United Arab Emirates” [2].

**Table:** Specifications Table

Subject	Earth and Planetary Sciences (Geophysics)
Specific subject area	Seismic hazard assessment
Type of data	Tables, Figures
How data was acquired	Earthquake raw data was compiled from the following publications and bulletins: Ambraseys and Melville [3], Ambraseys et al. [4], Aldama-Bustos [5], El-Hussain et al. [6], Shahvar et al. [7], Deif et al. [8], the International Institute of Earthquake Engineering and Seismology (IIEES) [9], the Iranian Seismological Center (IRSC) [10], the regional ISC-GEM Global Instrumental Earthquake catalog [11], EHB catalog [12], the International Seismological Center (ISC) Bulletin [13], and the United States Geological Survey (USGS) through the National Earthquake Information Center (NEIC) [14]. Focal-mechanisms data were obtained from the following published sources: the Global Centroid Moment Tensor (GCMT) catalog [15], the Zurich Institute of Technology (ZUR-RMT) catalog [16], the ISC [13], and the Iranian IIEES [9] bulletins, as well as other specific publications (e.g. Ref. [17], and [18]).
Data format	Raw, Analysed
Parameters for data collection	Several parameters were considered during the data collection. Examples of such parameters include geographic coordinates (longitudes and latitudes), start and end dates, minimum and maximum depth values, minimum and maximum magnitude values, and reported magnitude type, among others.
Description of data collection	Data were collected from several sources including international, regional, and local bulletins and publications. A minimum magnitude equal to 4.0 was considered on any reported magnitude scale within the investigated coordinates and during the compilation. Both historical and instrumental earthquakes were collected in this dataset.
Data source location	Country: United Arab Emirates (UAE) Latitude and longitude: a spatial region spanning latitudes between 21° and 31°N and longitudes between 47° and 66°E.
Data accessibility	The data files are available on a public repository Repository name: Mendeley Data, v1. Data identification number: 10.17632/t4ck8gp3jh.1 Direct URL to data: https://doi.org/10.17632/t4ck8gp3jh.3 [1]
Related research article	Authors names: Rashad Sawires, José A. Peláez, Mohammad AlHamaydeh, and Jesús Henares Title: A State-Of-The-Art Seismic Source Model for the United Arab Emirates Journal: Journal of Asian Earth Sciences DOI: 10.1016/j.jseaes.2019.104063 [2]

**Table:** 

**Value of the Data** • An updated and unified Poissonian earthquake catalog such as the one presented herein is greatly needed for any region in order to study the spatial and temporal distributions of the regional and local seismic activity. • Earthquake datasets in this article can be used to estimate the seismicity recurrence parameters (a-value, b-value, maximum expected magnitude) that serve as direct inputs for a seismic hazard assessment. • Focal-mechanism solutions serve as very useful indicators for the seismotectonic situation and the stress regime of a certain region. • Both earthquake and focal mechanism catalogs can be incorporated during the definition and characterization of the potential seismic sources surrounding a certain region. • Definition of a representative and reliable seismic source model based on such earthquake and focal mechanism catalogs is the most crucial element necessary to achieve appropriate estimates of seismic hazard and risk. It also facilitates establishing a unified seismic action representation in the building codes for a particular geographical region.

## 1 Data

**Fig. 1:**
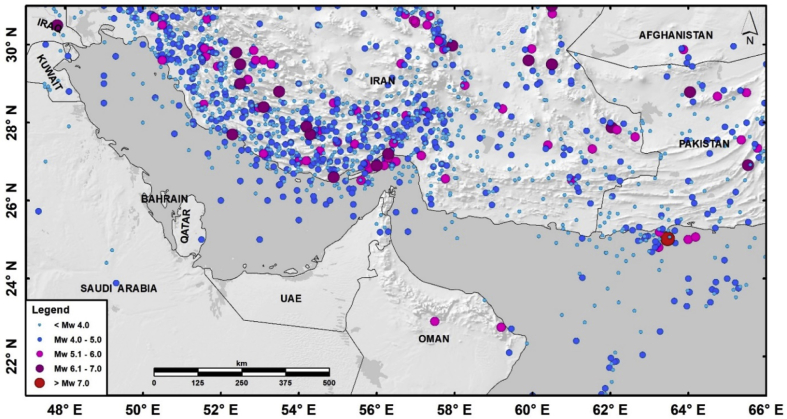
Earthquake dataset (658–2019) spatial distribution for the UAE and its surroundings [1]. Seismicity size is relative to the moment magnitude. All used coordinates are in the 1984 World Geodetic System (GCS-WGS84).

The first dataset is an updated Poissonian and unified earthquake catalog for the UAE that can be downloaded from the Mendeley Data repository [1] (see Fig. 1). The raw data was compiled from several local, regional and international sources [e.g. 3–14]. Several magnitude-conversion relationships have been applied to the compiled catalog to convert the reported magnitudes into a unified moment magnitude scale. Dependent earthquakes (e.g. earthquake swarms and aftershocks) were identified and removed through the declustering process. The final analysed earthquake catalog comprises of 1464 mainshocks spanning the time period from 658 to 2019, and the spatial region from 21° to 31°N latitudes, and from 47° to 66°E longitudes [1]. This dataset is a Microsoft Excel worksheet consisting of three sheets. The first two sheets contain a total of 1464 earthquakes (43 pre-1900 earthquakes in the first sheet, and 1421 post-1900 earthquakes in the second one) organized into 24 columns; each row describes a single main earthquake while each column describes the related parameters. The third sheet includes the codes, sources, and references that were used during the compilation. In the case that there is no value for any particular parameter, a zero was placed instead. The definition of the different parameters (columns) mentioned in the first and the second sheets are the following:

A: YEAR; B: MONTH; C: DAY: date type variables indicating the date for each earthquake.
D: HOUR; E: MINUTE; F: SECOND: date type variables indicating the time for each earthquake.
G: LONGITUDE; H: LATITUDE: double type variables (three decimal digits) indicating the location (longitude and latitude) for each event.
I: DEPTH: double type variable (one decimal digit) indicating the depth of each individual earthquake.
J: Mb; L: Ms1; N: Ms2; P: Mw; R:MD; T: ML; V:MN: double type variables indicating the reported magnitudes (one decimal digit) for the included earthquakes; they are as follows: Mb (Body-wave magnitude), Ms1 and Ms2 (Surface-wave magnitudes), Mw (Moment magnitude), MD (Duration magnitude), and ML and MN (Local magnitudes).
K, M, O, Q, S, U, W (Code): numbers representing the reference(s) for each previously-mentioned parameters (K for Mb, M for Ms1, O for Ms2, Q for Mw, S for MD, U for ML, and W for MN).
X: Mw*: double type variable (one decimal digit) indicating the final/equivalent computed moment magnitude for each earthquake included in the final catalog for the United Arab Emirates.

**Fig. 2:**
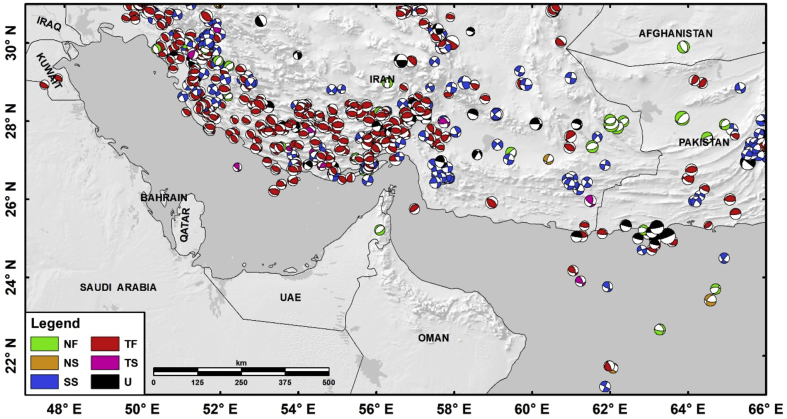
Spatial distribution of the focal-mechanism solutions dataset (1923–2015) in and around UAE [1]. Different colours refer to different faulting mechanisms; Green: pure normal-faulting (NF); Orange: normal-faulting with strike-slip component (NS); Blue: pure strike-slip faulting (SS); Red: pure reverse-faulting (TF); Pink: reverse-faulting with strike-slip component (TS); and Black: undefined faulting (U). Focal sizes are relative to the moment magnitude. All used coordinates are in the 1984 World Geodetic System (GCS-WGS84).

The second Microsoft Excel file in the Mendeley Data repository includes the focal mechanism solutions dataset [1]. It incorporates the final compiled focal mechanism solutions for the United Arab Emirates (see Fig. 2). This dataset comprises of 583 focal mechanism solutions spanning the same previously mentioned spatial region (21°–31°N latitudes, and 47° to 66°E longitudes) within the time frame from 1923 to 2015 [1]. Parameters of the two nodal planes (strike, dip, rake) for each earthquake solution are included in the dataset. This dataset was gathered from several published sources and bulletins (e.g. Refs. [9,[13], [14], [15], [16], [17], [18]], among others). This Microsoft Excel file consists of two sheets. The first sheet contains the data, while the other contains the references for the sources used in the compilation. The first sheet is comprised of 18 columns and 584 rows. Each row describes a single focal mechanism solution for each earthquake while each column describes the related parameters. In the case that there is no value for any particular parameter, a zero was placed instead. The parameters included in this Microsoft Excel sheet are the following:

A: YEAR; B: MONTH; C: DAY: date type variables indicating the date for each earthquake.
D: HOUR; E: MINUTE; F: SECOND: date type variables indicating the time for each earthquake.
G: LONGITUDE; H: LATITUDE: double type variables (three decimal digits) indicating the location (longitude and latitude) for each event.
I: DEPTH: double type variable (one decimal digit) indicating the depth of each individual earthquake.
J: Mw: double type variable (one decimal digit) indicating the final considered moment magnitude for each earthquake included in the catalog.
L: Strike 1; M: Dip 1; N: Rake 1; O: Strike 2; P: Dip 2; Q: Rake 2: these columns represent the two nodal planes for the focal mechanism solution for each earthquake; each column contains a number for each mentioned individual parameter (strike, dip, and rake angles).
K: Code; R: Code: these two columns refer to the source of the moment magnitude value and the focal mechanism solution parameters (strike, dip, and rake angles), respectively.

## 2 Experimental design, materials, and methods

A spatial region spanning longitudes between 47° and 66°E and latitudes between 21° and 31°N was considered during the dataset compilation. The dataset was mainly compiled from the following bulletins and publications: Ambraseys and Melville [3], Ambraseys et al. [4], Aldama-Bustos [5], the International Institute of Earthquake Engineering and Seismology (IIEES) [9], the Iranian Seismological Center (IRSC) [10], global instrumental ISC-GEM catalog [11], EHB catalog [12], the International Seismological Center (ISC) Bulletin [13], and the United States Geological Survey (USGS) through the National Earthquake Information Center (NEIC) [14]. Moreover, specific publications were also considered in the compilation (e.g. Refs. [[6], [7], [8]]). Magnitudes were recorded using different scales (moment “Mw”, surface-wave “Ms”, body-wave “mb”, and local “ML and MN” magnitudes) in the primary catalogs. Some magnitude conversion scaling relationships proposed by Shahvar et al. [7] for the Zagros and Central Iran regions were applied to unify the catalog in terms of the moment magnitude scale. Dependent earthquakes were identified and excluded from the unified compiled catalog by means of a declustering process using the algorithm by Gardner and Knopoff [19]. The final earthquake catalog includes 1464 mainshocks spanning years 685–2019. Figure (1) shows the spatial distribution of the final declustered catalog [1].

Focal-mechanism solutions for the surroundings of the UAE (between 47° and 66°E longitude and 21° to 31°N latitude) were mainly obtained from the following published sources and bulletins: the Global Centroid Moment Tensor (GCMT) catalog [15], the Zurich Institute of Technology (ZUR-RMT) catalog [16], the ISC [13], and the Iranian IIEES [9] bulletins, as well as other specific publications (e.g. Refs. [17,18]). A total number of 583 solutions spanning the time period of 1923–2015 were included [1]. These solutions have been depicted (Fig. 2) according to the classification of faulting mechanism given by Frohlich [20].

The related article has been published in the Journal of Asian Earth Sciences under the title “A State-Of-The-Art Seismic Source Model for the United Arab Emirates”. https://doi.org/10.1016/j.jseaes.2019.104063 [2].
